# Successful interventional management of abdominal compartment syndrome caused by blunt liver injury with hemorrhagic diathesis

**DOI:** 10.1186/1749-7922-9-20

**Published:** 2014-03-22

**Authors:** Hiroyuki Tokue, Azusa Tokue, Yoshito Tsushima

**Affiliations:** 1 Department of Diagnostic and Interventional Radiology, Gunma University Hospital, 3-39-22 Showa-machi, Maebashi, Gunma 371-8511, Japan

**Keywords:** Abdominal compartment syndrome, Transcatheter arterial embolization, N-butyl cyanoacylate

## Abstract

We report that a case of primary abdominal compartment syndrome (ACS), caused by blunt liver injury under the oral anticoagulation therapy, was successfully treated. Transcatheter arterial embolization (TAE) was initially selected, and the bleeding point of hepatic artery was embolized with N-Butyl Cyanoacylate (NBCA). Secondary, percutaneous catheter drainage (PCD) was performed for massive hemoperitoneum. There are some reports of ACS treated with TAE. However, combination treatment of TAE with NBCA and PCD for ACS has not been reported. Even low invasive interventional procedures may improve primary ACS if the patient has hemorrhagic diathesis or coagulopathy discouraging surgeon from laparotomy.

## Background

Abdominal compartment syndrome (ACS) is a life-threatening disorder, resulting when the consequent abdominal swelling or peritoneal fluid raises intraabdominal pressures (IAP) to supraphysiologic levels. ACS is defined as IAP above 20 mmHg together with a new organ failure. The recommended treatment is initially medical while surgical decompression is indicated only when medical therapy fails [1-3]. However, it is hardly possible to achieve operation without any complications on ACS, and more difficult in the aged patients or hemorrhagic diathesis. We report that a case of primary ACS, caused by blunt liver injury under the oral anticoagulation therapy, was successfully treated with interventional techniques. Additionally, we reviewed the previous reports of ACS treated with transcatheter arterial embolization (TAE). It may be considered as an alternative to surgical intervention for an ACS.

## Case presentation

**Figure 1:**
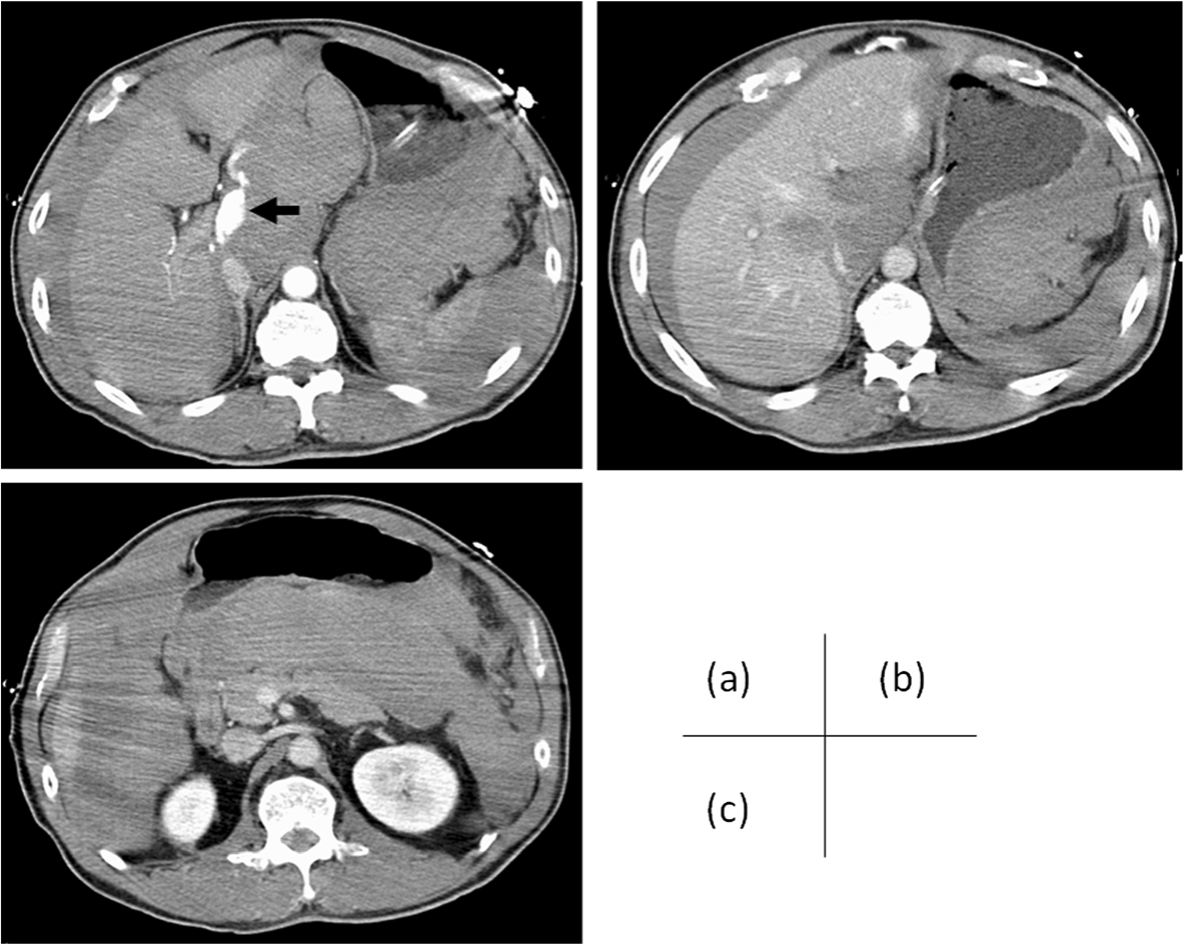
**A 71-year-old man was admitted to emergency unit for abdominal trauma due to traffic accident. (a)** CT showed that contrast material extravasation was in the hepatic hilum on arterial phase (arrow), and **(b)** an uncovered laceration extended over segments 1, 4 and 8 of the liver with massive hemoperitoneum. **(c)** CT scan at level at which left renal vein crosses aorta shows hemopritoneum. The ratio of anteroposterior-to-transverse diameter was equal to 1:0.76.

**Figure 2:**
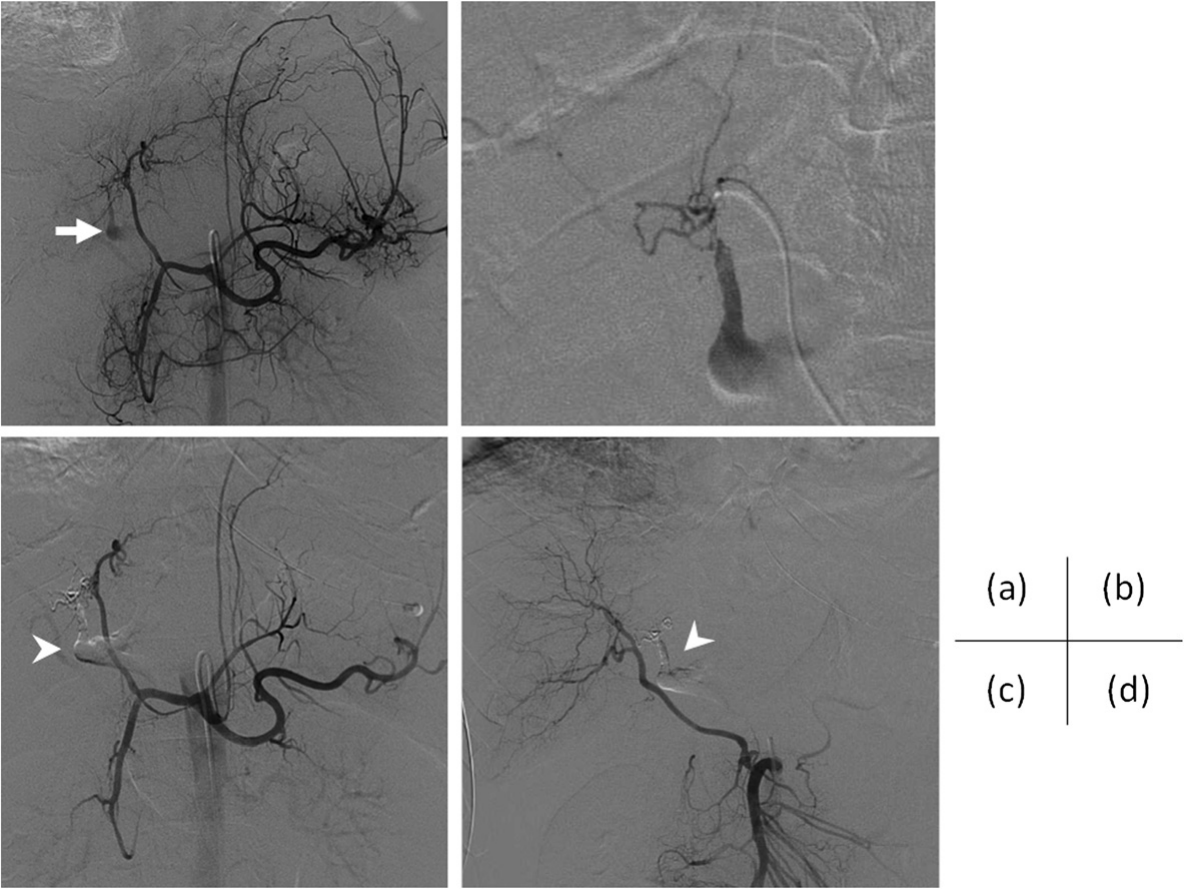
**The images of digital subtraction angiography (DSA).** The right hepatic artery arose from the superior mesenteric artery (SMA). **(a)** Celiac arteriography demonstrated contrast material extravasation from the left hepatic arterial branch (arrow). **(b)** Super selective DSA was confirmed leakage of the left hepatic aiterial branch. **(c)** After transcatheter arterial embolization, DSA of the celiac artery and **(d)** SMA did not demonstrate extravasation. Filled N-Butyl Cyanoacylate (NBCA) and Lipiodol were seen (arrowheads).

A 71-year-old man was admitted to emergency unit for abdominal trauma due to traffic accident. His consciousness was unclear and shock index was 1.8 (blood pressure, 70/39 mm Hg; pulse 125 beats/min). The electrocardiogram showed atrial fibrillation. His chest radiography showed markedly elevated diaphragms. The abdomen was distended, there were decreased sounds, and it was diffusely tender. Laboratory findings were as follows: hemoglobin 6.7 g/dL; international normalized ratio (INR) 3.2; because he was on the oral anticoagulation therapy for aterial fibrillation with warfarin and asprin. Arterial blood gas analysis revealed acute respiratory failure with a pH value of 7.344, PaO2 of 61.5 torr, PaCO2 of 49.0 torr under 5 L/min of oxygen supplementation by face mask. His urinary bladder pressure equal to intraabdominal pressures (IAP) was 26 cmH2O. He became hemodynamically unstable with hypotension. Transfusion of fresh frozen plasma and packed red blood cells was followed by a fluid overload and vitamin K. And he was placed on ventilator. Ultrasonography detected a hemoperitoneum and liver laceration. Enhanced computed tomography (CT) showed that contrast material extravasation was in the hepatic hilum on arterial phase (Figure 1a), and an uncovered laceration extended over segments 1, 4 and 8 of the liver with massive hemoperitoneum (Figure 1b,c). There were associated several rib fractures in the right upper quadrant and mild right hemothorax. Finally, we diagnosed as primary ACS. However, surgeons hesitated to perform laparotomy because of his hemorrhagic diathesis, therefore TAE was initially selected. The celiac artery was quickly cannulated with a 5-Fr shephered hook catheter (Clinical Supply Co. Ltd., Gifu, Japan). Digtal subtraction angiography (DSA) of the celiac artery demonstrated the perforated left hepatic arterial branch with exravasation (Figure 2a). The right hepatic artery was replaced on the superior mesenteric artery without extravasation. 2.0-Fr coaxial microcatheter (Progreat, Terumo Corp., Tokyo) was advanced nearby the bleeding point of the left hepatic arterial branch using a 0.014-in. microguidwire (Transend EX, Boston Scientific Corp., Watertown, MA, USA) (Figure 2b). Embolizaion was performed using mixtures of 0.1 mL of N-Butyl Cyanoacylate (NBCA) and 0.5 mL of Lipiodol. After TAE, DSA did not demonstrate extravasation (Figure 2c,d) and the patient became hemodynamically stable. Under ultrasonographic guidance, we inserted a 10.2-Fr pigtail drainage catheter (Cook Inc., Bloomington, IN, USA) into the right paracolic gutter using Seldinger’s technique. At the same time, IAP measured with the pigtail catheter was 30 cmH2O. About 3.2 L of intra-abdominal blood was evacuated through the pigtail catheter for the next two hours. IAP dropped to 12 cmH2O. He was discharged from the hospital without any major complications on 32 days after TAE.

## Discussion

ACS is a life-threatening condition resulting when the consequent abdominal swelling or peritoneal fluid raises intraabdominal pressures (IAP) to supraphysiologic levels, in massive abdominal hemorrhage, ascites, pancreatitis, ileus, as above [1-3]. At the World Congress of ACS in 2004, the World Society of Abdominal Compartment Syndrome, ACS is defined as an IAP above 20 mmHg with evidence of organ dysfunction/failure [4,5]. In our case, respiratory failure had been revealed. Increased IAP causes venous stasis and arterial malperfusion of all intra-and extra-abdominal organs, resulting in ischemia, hypoxia and necrosis. In parallel, respiratory, cardiocirculatory, renal, intestinal and cerebral decompensation can be seen.

Recently, ACS is divided to three types [4,5]. Primary (postinjury) ACS, applied to our case, is a condition associated with injury or disease in the abdomino-pelvic region that frequently requires early surgical or interventional radiological intervention. Total body shock and subsequent reperfusion with intestinal edema and a tightly packed and closed abdomen increase abdominal pressure.

Secondary ACS refers to conditions that do not originate from the abdomino-pelvic region. The typical injury patterns are penetrating heart, major vessel, or extremity vascular trauma associated with profound shock and subsequent massive resuscitation resulting in whole-body ischemia or reperfusion injury. Recurrent ACS represents a redevelopment of ACS symptoms following resolution of an earlier episode of either prmary or secondary ACS.

Radiologically, Pickhardt et al. [1] described increased ratio of anteroposterior-to-transverse abdominal diameter over 0.8 on CT. However, Zissin [6], reported that valuable peritoneal diseases may increase this ratio without ACS, and Laffargue et al. [7] revealed that the ratio of anteroposterior-to-transverse abdominal diameter was under 0.8 in primary ACS. In our case, the ratio of anteroposterior-to-transverse diameter on CT was equal to 1:0.76 (Figure 1c).

We suppose that ACS is not always completed on that time when the CT is performed to the patient with active intraabdominal hemorrhage. Therefore, we should make a diagnosis of ACS as soon as possible; the most useful and simple examination is measurement of IAP, substituted by urinary bladder pressure.

ACS is generally required surgical decompression, whereas unaccustomed surgeons hesitate to perform laparotomy, because of perioperative high mortality rate, long staying at the intensive care unit, reoperation, and late complications including incisional hernia, gastrointestinal and pancreatic fistulas, abscess, polyneuropathy, psychic disorders, as above [1]. Additionally, our patient was on hemorrhagic diathesis with the oral anticoagulation therapy for atrial fibrillation, and attended with suspicious disseminated intravascular coagulation due to massive hemorrhage. But it wcxxas expected that the major vascular leakage was only in the hepatic arterial branch without any bowel perforation on the contrast-enhanced CT, so we performed interventional procedure. NBCA was the most appropriate embolic agent of TAE for our case with hemorrhagic diathesis, because it does not depend on the coagulation process for its therapeutic effect [8]. There are some reports of ACS treated with TAE [9]. However, combination treatment of TAE with NBCA and percutaneous catheter drainage (PCD) for ACS has not been reported (Table 1). We suggest that initial hemostasis by transcatheter arterial embolization is a safe, effective treatment method for abdominal compartment syndrome with active arterial bleeding in a patient undergoing anticoagulation.

**Table 1:** The characteristics of the reported cases of abdominal compartment syndrome treated with transcatheter arterial embolization

**Author**	**N**	**Clinical presentation**	**Embolized artery**	**Embolic material**	**Subsequent treatment**
Letoublon [9]	14	Blunt hepatic trauma	Hepatic artery	NS	Decompressive laparotomy or laparoscopy
Won [10]	1	Retroperitoneal hemorrhage	Internal iliac artery	Gelatin sponge, coil, lipiodol	Decompressive laparotomy
Pena [11]	1	Splenomegaly	Splenic artery	PVA	Nothing
Monnin [12]	7	Blunt hepatic trauma	Hepatic artery	Gelatin sponge, coil	Decompressive laparotomy
				Trisacryl gelatin microsphere	
Hagiwara [13]	1	Pelvic flactures	Super gluteal artery	Gelatin sponge	Repeat TAE, decompressive laparotomy
Isokangas [14]	5	Retroperitoneal hemorrhage	Lumbar artery (N = 4)	Gelatin sponge, PVA, coil	Surgical decompreesion (N = 4)
			Medial rectal artery (N = 1)		US guided drainage (N = 1)
Tokue (present)	1	Blunt hepatic trauma	Hepatic artery	NBCA, lipiodol	US guided drainage

N: number of patients, NS: not shown, PVA: polyvinyl alcohol, NBCA: N-Butyl Cyanoacylate, US: ultrasonography.

The decompression is simultaneously essential to hemostasis for the treatment of primary ACS. There are some randomized controlled trials for ACS (Table 2) [31]. However, there have been no randomized controlled trials about which is better, PCD or decompressive laparotomy. PCD is easy and minimal invasive procedure compared with surgical decompression, and allows us to measure IAP. But it is not appropriate to perform catheter drainage for the patients with widespread peritonitis or bowel injury. When a heavy clot burden cannot be drained satisfactorily via catheter, we should transfer to decompressive laparotomy.

**Table 2:** Characteristics of the randomized controlled trials on IAP, IAH, and ACS

**Author**	**N**	**Study population**	**Intervention**	**Control**	**Main conclusion**
Celik [15]	100	Patients undergoing elective	5 different IAP levels; 8, 10,	NA	No effect of IAP levels on gastric
		Laparoscopic cholecystectomy	12, 14, and 16 mm Hg		intramucosal pH
Basgul [16]	22	Patients undergoing elective laparoscopic cholecystectomy	Low IAP level (10 mm Hg)	High IAP level (14Y15 mm Hg)	Less depression of immune function (expressed as interleukin 2 and 6) in the low IAP group
O’Mara [17]	31	Burn patients (>25% TBS with inhalation injury or >40% TBS without)	Plasma resuscitation	Crystalloid resuscitation	Less increase in IAP and less volume requirement in plasma-resuscitated patients
Sun [18]	110	Severe acute pancreatitis patients	Routine conservative treatment combined with indwelling catheter drainage	Routine conservative treatment	Lower mortality, lower APACHE II scores after 5 d and shorter hospitalization times in intervention group
Bee [19]	51	Patients undergoing emergency laparotomy requiring temporary abdominal closure	Vacuum-assisted closure	Mesh closure	No signification differences in delayed fascial closure or fistula rate
Karagulle [20]	45	Patients undergoing elective laparoscopic cholecystectomy	3 different IAP levels; 8, 12, and 15 mm Hg	NA	Similar effects on pulmonary function test results
Zhang [21]	80	Severe acute pancreatitis patients	Da-Cheng-Qi decoction enema and sodium sulphate orally	Normal saline enema	Lower IAP levels in intervention group
Ekici [22]	52	Patients undergoing elective laparoscopic cholecystectomy	Low IAP level (7 mm Hg)	High IAP level (15 mm Hg)	More pronounced effect of high IAP on QT dispersion
Joshipura [23]	26	Patients undergoing elective laparoscopic cholecystectomy	Low IAP level (8 mm Hg)	High IAP level (12 mm Hg)	Decrease in postoperative pain and hospital stay, and preservation of lung function in low pressure level group
Mao [24]	76	Severe acute pancreatitis patients	Controlled fluid resuscitation	Rapid fluid resuscitation	Lower incidence of ACS in controlled fluid resuscitation group (i.a.)
Yang [25]	120	Severe acute pancreatitis patients	Colloid plus crystalloid resuscitation	Crystalloid resuscitation	Decline of IAP was significant higher in crystalloid plus colloid group
Celik [26]	60	Patients undergoing elective laparoscopic cholecystectomy	3 different IAP levels; 8, 12 and 14 mm Hg	NA	No effect of IAP level on postoperative pain
Chen [27]	60	ICU patients with multiorgan failure	Tongfu Granule	Placebo	Decreased IAP in intervention group
			(Traditional Chinese medicines)		
Agarwal [28]	190	Patients undergoing emergency laparotomy	Reinforced tension line sutures	Continuous suturing	No difference in IAP but increased incidence of fascial dehiscence in continuous suture group
Du [29]	41	Severe acute pancreatitis patients	Hydroxyethyl starch resuscitation	Ringer’s lactate resuscitation	Lower incidence of IAH and reduced use of mechanical ventilation in intervention group
Topal [30]	60	Patients undergoing elective laparoscopic cholecystectomy	3 different IAP levels; 10, 13, and 16 mm Hg	NA	No differences on thromboelastography

N: number of patients, APACHE: Acute Physiology And Chronic Health Evaluation, NA: not applicable/available; TBS: Total body surface area, IAP: intra-abdominal pressure, IAH: intra-abdominal hypertension, ACS: abdominal compartment syndrome.

## Conclusions

In summary, we described the case of primary ACS caused by blunt liver injury. Interventional procedures may improve primary ACS if the patient has hemorrhagic diathesis or coagulopathy discouraging surgeon from laparotomy, limited vascular injury, and no obvious peritonitis.

## Consent

Written informed consent was obtained from the patient for publication of this Case report and any accompanying images. A copy of the written consent is available for review by the Editor of this journal.

## Competing interests

The authors declare that they have no competing interests.

## Authors’ contributions

All authors read and approved the final manuscript.
